# Influence of grinding on the nutritive value of peas for ruminants: comparison between in vitro and in situ approaches

**DOI:** 10.1002/fsn3.90

**Published:** 2014-04-04

**Authors:** Sylvie Giger-Reverdin, Chiraze Maaroufi, Patrick Chapoutot, Corinne Peyronnet, Daniel Sauvant

**Affiliations:** 1 INRA, UMR791 Modélisation Systémique Appliquée aux RuminantsF-75005, Paris, France; 2 AgroParisTech, UMR 791 Modélisation Systémique Appliquée aux RuminantsF-75005, Paris, France; 3 UNIP11 rue de Monceau, CS 60003, F-75008, Paris, France

**Keywords:** Grinding, in situ method, in vitro method, nutritive value, pea, ruminant

## Abstract

In ruminant nutrition, peas are characterized by high protein solubility and degradability, which impair its protein value estimated by the official in situ method. Grinding can be used as a technological treatment of pea seeds to modify their nutritional value. The aim of this study was to compare the in situ method with an in vitro method on the same pea either in a coarse pea flour form (PCF) or in a ground pea fine flour form (PFF) to understand the effect of grinding. Both forms were also reground (GPCF and GPFF). PCF presented a lower rate of in vitro degradation than PFF, and more stable fermentation parameters (pH, ammonia, soluble carbohydrates) even if gas production was higher for the PCF after 48 h of incubation. In situ dry matter and protein degradation were lower for PCF than those for PFF; these differences were more marked than with the in vitro method. Reground peas were very similar to PFF. The values for pea protein digestible in the intestine (PDI) were higher for PCF than those for PFF. This study points out the high sensitivity of the in situ method to grinding. The study needs to be validated by in vivo measurements.

## Introduction

Pea production has greatly increased during the last few decades in order to reduce the dependency of the European Union on imported protein-rich feeds. Peas are also very interesting from a nutritional point of view as a “dual-purpose” feed because, in addition to its protein content, it has an energy value close to that of cereals due to its high starch content (Sauvant et al. 2004). It is then of importance to estimate with accuracy the nutritive value of this feed. The in situ technique is generally adopted as the standard method to characterize rumen degradability of protein, and thus protein values of feeds (Madsen 1985; Vérité et al. 1987; AFRC 1992; Tamminga et al. 1994). Several studies pointed out that the protein value of peas could be underestimated by this technique (Gatel 1995). Indeed, the truly digestible proteins in the small intestine (PDI) content (Jarrige et al. 1978) of pea is underestimated (Gatel 1995) because of its highly soluble and degradable protein fraction (Aguilera et al. 1992; Solanas et al. 2008). Moreover, this could lead to excessive urinary waste of nitrogen (Michalet-Doreau 1992; Guedes and da Silva 1996). Several technological treatments have been proposed to modify the degradation of protein by microorganisms in the rumen. Among them, grinding has been shown to be able to modify the protein degradability of ingredients rich in protein such as pea seeds (Freer and Dove 1984; Michalet-Doreau and Cerneau 1991; Lykos and Varga 1995; Bayourthe et al. 2000) because it is a way to control particle feed size, and thus, the availability of cellular constituents for the rumen flora (Brennan et al. 1976). The estimation of the PDI value of a feed after grinding poses a methodological problem as the standardization of the in situ method requires grinding the feed with a 0.8 mm screen (Michalet-Doreau et al. 1987). It is well known that results obtained with the nylon bag method depend on the relative sizes of feed particles and pore sizes of the bag (Huntington and Givens 1995).

Maaroufi et al. (2009) pointed out that the rate of N degradation in vitro of the various subfractions of ground pea flour increased when their particle size decreased. However, there was a confounding effect between the chemical composition of the subfractions and their particle size. The aim of this study was to compare data obtained with the same in vitro method (Menke et al. 1979) which is very often used to estimate the energy value of feeds and the official in situ method for the PDI system (Michalet-Doreau et al. 1987) in order to estimate the nutritive value of raw pea either in a coarse form or in a ground form.

## Material and Methods

### Experimental plant material

Two grinding techniques were applied to pea seeds (*Pisum sativum*, Baccara variety) in order to obtain different particle size distributions. The pea coarse flour (PCF) was obtained with a crushing roller (Socam, ATEM Industrie, Saint-Barthelemy d'Anjou, France) equipped with two cylinders. The spacing between the rollers was 2.5 mm and the rate of throughput was 300 kg h^−1^. The pea fine flour (PFF) was produced by grinding the seeds with a hammer mill, fitted with a 2 mm screen at a rate of 800 kg h^−1^ (Promill Type B4 C; Promill, Dreux, France, 1990). The particle size distribution was determined with a laboratory siever (Bühler MLU 300; Bühler-Miag, Uzwil, Switzerland) using a set of 12 woven-wire cloth sieves (sieve opening sizes from 315 to 8000 *μ*m for PCF and from 80 to 2500 *μ*m for PFF). Sieving lasted for 15 min and was carried out in duplicate. Because of the grinding methodology for the in vitro and in situ studies, both PCF and PFF flours were reground through a screen of 1 mm aperture (named GPCF and GPFF, respectively). Thus, the four flours were tested in vitro and in situ. The PCF and PFF flours were analyzed by standard methods for dry matter, ISO (1983) ash, ISO (1978) crude protein (CP), ISO (1997), and starch ISO (2004). Cell wall content was estimated by the neutral detergent fiber (NDF) method of Van Soest and Wine (1967) modified by Giger et al. (1987). Lignocellulose or acid detergent fiber and lignin were obtained using a sequential approach on the NDF residue (Giger et al. 1987). Furthermore, some physical properties were measured: granulometric profile with the determination of median diameter, specific surface area, and apparent density. The methods used have been previously described (Maaroufi et al. 2000). The original and reground flours were also analyzed in duplicate for their initial pH and buffering capacity with 2 N acetic acid (Giger-Reverdin et al. 2002).

### In vitro gas test study

Pea flour fermentation was measured using an adaptation of the gas test or HFT (Hohenheimer Futterwert Test)method (Menke and Steingass 1988) as described by Maaroufi et al. (2009). Rumen fluid was obtained from two ruminally fistulated dry cows fed the same diet (70:30 grass hay: concentrate ratio on a DM basis) as the one used for the protein in situ degradability (Michalet-Doreau et al. 1987).

In the first in vitro trial, the flours and their reground samples (PCF, PFF, GPCF, and GPFF) were incubated in syringes for up to 48 h. Gas volumes were recorded at all the times of fermentation (1, 2, 4, 6, 8, 12, 24, 32, and 48 h), and one-third of the syringes was emptied after 2, 8, and 48 h. In the second in vitro trial, the focus was on the differences between the two original flours PCF and PFF during the short- and medium-term incubations (up to 24 h): some syringes were emptied at 1, 2, 4, 6, 8, 12, and 24 h after measurement of gas production. When a syringe was emptied, pH was measured immediately after collection using a pH meter fitted with a glass electrode. Samples were then acidified with trichloroacetic acid (25 g L^−1^) to inhibit microbial activity. An aliquot was refrigerated at 4°C before the analysis of ammonia (NH_3_) with the method described by Weatherburn (1967) using an Auto analyzer (Technicon, Oise, France). Another aliquot was stored at −20°C until the determination of soluble carbohydrates with the same Auto analyzer (Technicon) using a nonspecific method adapted from Brown and Boston (1961). Results were expressed as glucose equivalents.

In both trials, measurements were duplicated in two separate runs. Within each run of incubations, with sampling at each duration, the fine flour (PFF) and the reground ones (GPFF and GPCF) were replicated twice, and the coarse one (PCF), thrice, because it was more difficult to sample the coarse flour in a homogeneous manner. Two standards (hay and concentrate samples) were provided by the University of Hohenheim (Germany) and were those proposed by Menke and Steingass (1988). They were placed in each run to estimate the between-day variation due to the inoculum.

### In situ study

The in situ degradation of the four pea samples (PCF, PFF, GPCF, and GPFF) was measured according to the method proposed by Michalet-Doreau et al. (1987) but without any further grinding for the PCF and PFF samples. Samples (3 g DM) were placed in nylon bags of internal dimensions 6 × 11 cm and pore size of about 46 *μ*m. Three dry cows fitted with a rumen cannula received 7 kg DM (14% CP on a DM basis) in two equal meals at 9:00 and 17:00 h. All bags, except those of the 16 h duration, were introduced into the rumen at the same time, just before the morning feeding, and removed after 2, 4, 8, 24, and 48 h of incubation. The “16 h bags” were introduced before the afternoon feeding and removed the next morning with the 24 h bags. For each time and each feed, there were six replicates as each feed was tested with each of the three cows in two separate runs. Upon removal from the rumen, bags were immediately rinsed in cold water and washed three times for 10 min in a washing machine using cold water to suppress microbial action, dried at 60°C for 48 h, and weighed for the estimation of DM degradation. The test of Anscombe and Tukey (Snedecor and Cochran 1984) was performed on DM degradation values to detect abnormal data. This resulted in removal of 2.0% of the data.

The nitrogen content was determined on residues pooled within each feed and each incubation time. However, after 48 h of degradation, only for the PCF sample enough residue remained to be analyzed.

### Statistical analysis

Time patterns of gas production were calculated with the data obtained over the 48 h incubations from the first trial and corrected with the actual quantity of samples introduced and actual initial volume of inoculum in order to have the volume of gas produced by 200 mg DM of sample with 30 mL of inoculum. These patterns were fitted according to a Gompertz function (Huhtanen et al. 2008),


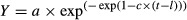


with *Y* as the cumulated gas production at time *t* (mL 200 mg^−1^ DM), *a* the potential total gas production, *c* the fractional rate of gas production (h^−1^), and *l* the assumed discrete lag phase before the onset of degradation. This model assumed that there was no gas production until *t* reached the value *l*. The adjusted values of parameters *a*, *c*, and *l* were obtained with a non linear (NLIN) procedure of SAS (2002). The gas production after 16 h and 40 min (GP16) was calculated, because this duration corresponds to the mean duration feed remains in the gastrointestinal tract assuming a fractional outflow rate *k*p = 0.06 h^−1^, as for in situ data (Michalet-Doreau et al. 1987).

The CP degradation was calculated using the DM degradation for each replicate and the mean CP content in the residue. The least square mean (LSMEANS) values of DM and CP degradation were fitted either with the monomolecular model used by Ørskov and McDonald (1979),


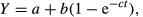
model 1

or by the modification of this model proposed by McDonald (1981),


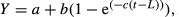
model 2

where *Y* is the degradation at time *t* in %, *a* the immediately soluble fraction (%), *b* the potentially degradable fraction (%), and *c* the relative rate of degradation of fraction *b* (h^−1^). The adjusted values of parameters *a*, *b*, and *c* were obtained with the NLIN procedure of SAS (2002). The effective degradability (ED) was calculated assuming that the outflow rate of particles (*k*p) was equal to 0.06 h^−1^ (Vérité et al. 1987) according either to the following formula for the first model (Ørskov and McDonald 1979),


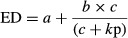


or for the second model (McDonald 1981) to: 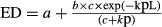.

Statistical analysis was carried out using PROC GLM (General Linear Model) of SAS 9.1. (SAS 2002). The model *Y*_*ij*_ = *μ* + *α*_*i*_ + *β*_*j*_ + *α*_*i*_ × *β*_*j*_ + *ε*_*ij*_ where *Y*_*ij*_ is the dependent variable, *μ* is the least square mean, *α*_*i*_ the feed effect, *β*_*j*_ the grinding effect and *α*_*i*_ × *β*_*j*_ the interaction between the first and second grinding. This was applied to initial pH, buffering capacity, the parameters of in vitro fermentation of the first trial, and the in situ degradation. As there is a between-day variation in the rumen juice (Menke and Steingass 1988; Getachew et al. 1998) in the in vitro and in situ trials, the incubation run was also included as a random variable, and in the in situ trial, a cow effect was also taken into account. The statistical comparisons of the different in vitro feed fermentation characteristics or in situ degradation were obtained by the LSMEANS procedure.

## Results

### Characteristics of the pea flours

Pea flours from the same origin presented quite similar values of CP and starch, but differed for some of the physical characteristics and for cell wall content (Table 1).

**Table 1:** The effect of grinding on chemical composition and physical characteristics of the same pea

	Pea coarse flour (PCF)	Pea fine flour (PFF)
Chemical composition (g kg^−1^ DM)		
Crude protein	244	237
Starch	572	548
Neutral detergent fiber	154	104
Acid detergent fiber	106	71
Acid detergent lignin	4	3
Ash	23	24
Physical characteristics		
Median diameter (*μ*m)	2825.4	344.6
Specific surface area (m^2^ g^−1^)	0.036	0.148
Apparent density (g cm^−1^)	1.445	1.455

PCF obtained with a crushing roller with a 2.5 mm space; PFF obtained with hammer mill with a 2 mm screen.

**Figure 1:**
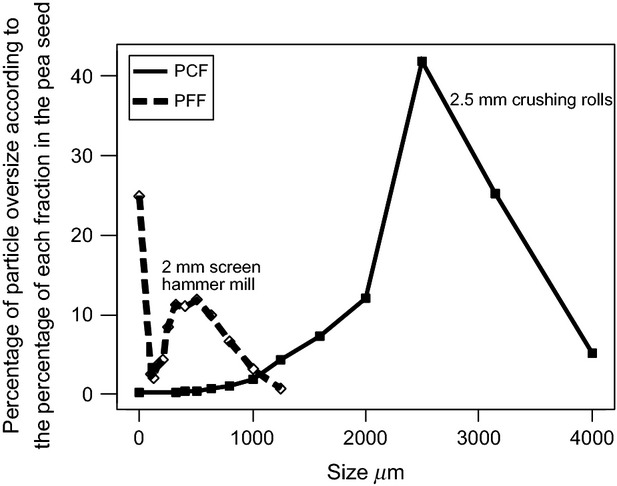
Pea particle size distribution of coarse and fine ground pea flours. PCF, pea coarse flour, obtained with a crushing roller with a 2.5 mm space; PFF, pea fine flour, obtained with hammer mill with a 2 mm screen.

The fine flour had a lower mean particle size estimated by the median diameter and a higher specific surface area (around four times) than the coarse one. Nevertheless, the apparent density was similar for both flours. The particle distribution showed a satisfactory discrimination between the two flours, as less than 10% of particles presented the same size (Fig. 1).

PFF had a lower initial pH, but a higher buffering capacity than the pea coarse one (PCF) (Table 2). There was no effect of the re-grinding process on initial pH. The coarse pea (PCF) had a lower buffering capacity than the ground coarse pea (GPCF), which was similar to both pea fine flours (PFF and GPFF).

**Table 2:** Initial pH and buffering capacity of the pea flours

	Pea grinding method					*P*-value		
		
	PCF	GPCF	PFF	GPFF	SEM	*F*	*G*	*F* × *G*
Initial pH	6.59^a^	6.57^a^	6.49^b^	6.48^b^	0.015	0.001	0.26	0.65
Buffering capacity	0.170^a^	0.533^b^	0.536^b^	0.555^b^	0.0068	0.001	0.001	0.001

Data are presented as LSMEANS ± SEM. PCF, pea coarse flour, obtained with a crushing roller with a 2.5 mm space; GPCF, PCF reground with a screen of 1 mm aperture; PFF, pea fine flour, obtained with hammer mill with a 2 mm screen; GPFF, PFF reground with a screen of 1 mm aperture; *F*, feed effect (coarse vs. fine); *G*, regrinding effect; *F* × *G*, interaction between feed and regrinding effects. Buffering capacity was determined with the method described by Giger-Reverdin et al. (2002). Mean values in the same row without a common superscript are significantly different at *P* < 0.05.

### HFT fermentation profiles

The blank “syringes,” filled with the *inoculum* without any substrate, had a mean gas production of 4 mL after 24 h of incubation. Due to these very low values, gas productions of pea samples were not corrected by the blank values. Moreover, the microbial activity in the “blank” syringes was not relevant to what occured in syringes containing feeds that bring energy and nitrogen to the medium and, in particular, microbial turnover did not start at the same incubation times (Cone et al. 1997; Williams 2000). Menke and Steingass (1988) proposed a correction factor, which was the mean value of correction factors for the standard hay and the standard concentrate. In this study, the correction factor for the standard hay gas volume was, for the four runs, 0.95 (±0.016) mL and that for the standard concentrate gas volume was 1.05 (±0.023) mL. Each of these correction factors was within the interval [0.9–1.1] as required by the authors of the method. Moreover, the values for the factor of correction on gas production from the standard samples were of 1.00 (±0.023) for the four runs. Given a value equal to 1 for this factor, the gas productions of pea samples were not corrected at all.

### First trial

#### Gas production

The PCF pattern of gas production differed from that of the three other flours (Table 3). Gas production of PCF was lowest during the first 12 h of incubation. However, after 24 h of incubation, the gas production of the four flours was similar, and afterward, the PCF gas production was higher than that of the other flours. At each incubation time, GPCF, PFF, and GPFF had similar gas productions.

**Table 3:** In vitro kinetics of the cumulative gas production with the pea flours (first gas test trial)

	Cumulative gas production (mL 200 mg^−1^ DM)					*P*-value			
		
Incubation time (h)	PCF	GPCF	PFF	GPFF	SEM	*F*	*G*	*F* × *G*	Run
1	2.6^a^	4.8^b^	4.6^b^	4.9^b^	0.15	<0.001	<0.001	<0.001	0.017
2	5.6^a^	9.2^b^	9.1^b^	9.4^b^	0.27	<0.001	<0.001	<0.001	<0.001
4	10.6^a^	18.5^b^	17.9^b^	18.5^b^	0.38	<0.001	<0.001	<0.001	<0.001
6	15.6^a^	33.2^b^	32.1^b^	33.0^b^	0.61	<0.001	<0.001	<0.001	<0.001
8	20.9^a^	48.0^b^	49.1^b^	49.7^b^	0.93	<0.001	<0.001	<0.001	0.007
12	35.4^a^	60.3^b^	63.4^b^	62.9^b^	1.51	<0.001	<0.001	<0.001	0.73
24	75.2^a^	71.3^a^	74.8^a^	74.1^a^	1.42	0.40	0.13	0.25	0.23
32	82.2^a^	75.8^b^	79.0^a,b^	78.9^a,b^	1.48	0.97	0.04	0.05	0.12
48	87.7^a^	79.5^b^	83.2^b^	82.8^b^	1.52	0.69	0.01	0.02	0.04

	Kinetic fitting parameters				RSE				
*a*	89.2^a^	75.6^b^	79.3^b^	78.8^b^	1.69	0.17	0.03	0.03	
*c*	0.123^a^	0.244^b^	0.243^b^	0.244^b^	0.0635	<0.001	<0.001	<0.001	
*l*	2 h 43 min^a^	1 h 7 min^b^	1 h 20 min^b^	1 h 13 min^b^	26 min	0.11	0.05	0.07	
GP16 (mL 200 mg^−^1 DM)	54.7^a^	71.1^b^	74.3^b^	74.1^b^	0.99	<0.001	<0.001	<0.001	

Data are presented as LSMEANS ± SEM. PCF, pea coarse flour, obtained with a crushing roller with a 2.5 mm space; GPCF, PCF reground with a screen of 1 mm aperture; PFF, pea fine flour, obtained with hammer mill with a 2 mm screen; GPFF, PFF reground with a screen of 1 mm aperture; *F*, feed effect (coarse vs. fine); *G*, regrinding effect; *F* × *G*, interaction between feed and regrinding effects; RSE, residual standard error of the model; GP16, gas production after 16 h and 40 min. Mean values in the same row without a common superscript are significantly different at *P* < 0.05.

**Figure 2:**
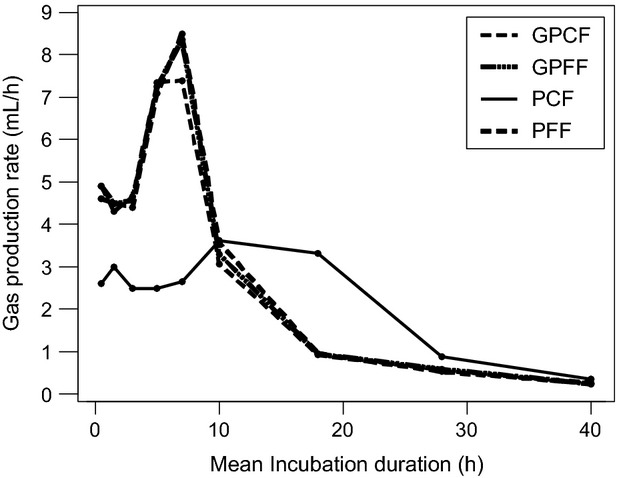
Rates of gas production for the different pea flours. PCF, pea coarse flour, obtained with a crushing roller with a 2.5 mm space; GPCF, PCF reground with a screen of 1 mm aperture; PFF, pea fine flour, obtained with hammer mill with a 2 mm screen; GPFF, PFF reground with a screen of 1 mm aperture.

The rate of gas production increased for the fine flours up to 8 h of incubation and decreased afterward (Fig. 2).

During the initial phase of fermentation (Table 3), the lag time was longer for PCF than that for PFF. The fractional degradation rate *c* of PCF was lower than that of PFF and this was also the case for gas production after 16 h and 40 min estimated by the cumulative gas production models. Reground samples (GPCF and GPFF) had similar values to those of PFF. This rate was lower for the PCF during the first part of incubation and then was always higher until the end of the experiment, with a kind of plateau between 8 and 24 h of incubation.

#### In vitro fermentation characteristics

The mixture of inoculum and substrate is referred to throughout as the medium. After 2 h of incubation, the PCF medium differed from the other media with a higher pH, a lower gas production, and a lower concentration of soluble carbohydrates (Table 4). Its ammonia concentration was the lowest but did not differ significantly from that of the GPFF medium. After 8 h of incubation, the PCF medium differed from that of the others for gas production and pH, which remained close to that of the initial medium (6.86). PCF had the lowest soluble carbohydrate concentration but was quite similar to that of others for ammonia concentration. After 48 h of incubation, PCF exhibited both the highest gas production and pH, and lowest concentration for ammonia but quite similar concentrations for soluble carbohydrates compared to the other flours. Although the pH of PCF medium was higher than that of the other samples, it decreased between 8 and 48 h of incubation contrary to the pH of the media containing fine flours, which remained fairly stable. The ammonia concentration increased during all the experiments regardless of the sample considered and was quite high at 48 h of incubation. The concentration of the soluble carbohydrates was very variable: it was quite high for GPFF after 2 h of incubation, then decreased at 8 h, and increased a second time after 48 h. There were similar, but less marked, evolutions for PFF and GPCF samples. For PCF, it increased during all the run.

**Table 4:** In vitro fermentation characteristics of the pea flours after 2, 8, or 48 h of incubation (first gas trial)

						*P*-value			
						
	PCF	GPCF	PFF	GPFF	SEM	*F*	*G*	*F* × *G*	Run
2 h of incubation									
Cumulative gas production (mL 200 mg^−1^ DM)	5.9^a^	9.3^b^	9.7^b^	9.2^b^	0.33	<0.001	<0.001	<0.001	<0.001
pH	6.88^a^	6.83^b^	6.84^b^	6.83^b^	0.012	0.12	0.01	0.08	0.01
Ammonia (mg L^−1^)	173^a^	178^b^	183^c^	176^a,b^	1.32	0.02	0.36	0.001	0.001
Soluble carbohydrates (mg L^−1^)	22.8^a^	47.5^b^	46.3^b^	56.5^c^	3.97	<0.001	<0.001	<0.001	<0.001
8 h of incubation									
Cumulative gas production (mL 200 mg^−1^ DM)	20.9^a^	48.8^b^	48.7^b^	50.7^c^	0.49	<0.001	<0.001	<0.001	0.004
pH	6.86^a^	6.65^b^	6.66^b^	6.64^b^	0.007	0.001	<0.001	<0.001	<0.001
Ammonia (mg L^−1^)	222^a,c^	212^b^	224^a^	218^c^	1.6	0.02	<0.001	0.17	0.01
Soluble carbohydrates (mg L^−1^)	28.2^a^	35.8^b^	34.3^c^	40.8^d^	0.41	<0.001	<0.001	0.20	<0.001
48 h of incubation									
Cumulative gas production (mL 200 mg^−1^ DM)	87.7^a^	79.5^b^	83.2^b^	82.8^b^	1.52	0.69	0.01	0.02	0.04
pH	6.74^a^	6.67^b^	6.66^b,c^	6.64^c^	0.0064	<0.001	<0.001	0.001	0.64
Ammonia (mg L^−1^)	415^a^	454^b^	469^b^	472^b^	11.7	0.03	0.15	0.22	0.01
Soluble carbohydrates (mg L^−1^)	67.0^a^	68.3^a,b^	68.0^a,b^	69.3^b^	0.57	0.10	0.05	1.00	<0.001

Data are presented as LSMEANS ± SEM. PCF, pea coarse flour, obtained with a crushing roller with a 2.5 mm space; GPCF, PCF reground with a screen of 1 mm aperture; PFF, pea fine flour, obtained with hammer mill with a 2 mm screen; GPFF, PFF reground with a screen of 1 mm aperture; *F*, feed effect (coarse vs. fine); *G*, regrinding effect; *F* × *G*, interaction between feed and regrinding effects. Mean values in the same row without a common superscript are significantly different at *P* < 0.05.

These results indicate that the PCF medium differed from the other samples, particularly during the first hours of incubation, and that the reground samples showed results very similar to PFF.

### Second trial

The results of the second trial were in agreement with those of the first one: the gas production of PCF was lower than that of PFF, during the first 12 h of incubation (Table 5). The pH of the PCF medium remained stable until 8 h of incubation and was similar to that of the PFF medium after 24 h of incubation.

**Table 5:** In vitro fermentation characteristics of pea flours in the short and medium terms (second gas test trial)

				*P*-value	
				
Incubation time (h)	PCF	PFF	SEM	*F*	Run
Cumulative gas production (mL 200 mg^−1^ DM)					
1	2.7^a^	4.6^b^	0.19	<0.001	0.05
2	4.6^a^	8.9^b^	0.47	<0.001	0.89
4	8.7^a^	17.5^b^	0.70	<0.001	0.01
6	13.6^a^	34.2^b^	1.05	<0.001	0.06
8	20.4^a^	52.4^b^	1.45	<0.001	1.00
12	38.7^a^	63.1^b^	3.00	<0.001	0.50
24	75.1^a^	78.1^a^	1.54	0.19	0.56
pH					
1	6.80^a^	6.79^a^	0.008	0.26	0.05
2	6.80^a^	6.79^a^	0.006	0.07	0.01
4	6.81^a^	6.76^b^	0.006	<0.001	0.01
6	6.80^a^	6.70^b^	0.010	<0.001	0.01
8	6.78^a^	6.64^b^	0.007	<0.001	0.19
12	6.74^a^	6.64^b^	0.013	0.001	0.40
24	6.62^a^	6.62^a^	0.001	0.90	0.11
Ammonia concentration (mg L^−1^)					
1	169^a^	171^a^	1.0	0.36	0.007
2	173^a^	181^b^	1.5	0.006	0.003
4	185^a^	199^b^	2.7	0.007	0.01
6	197^a^	206^b^	2.1	0.02	<0.001
8	210^a^	205^b^	1.6	0.06	0. 46
12	239^a^	222^b^	3.0	0.01	0.08
24	298^a^	296^a^	5.9	0.89	0.28
Soluble carbohydrates (mg L^−1^)					
1	27.6^a^	55.9^b^	0.98	<0.001	0.19
2	25.7^a^	49.6^b^	0.45	<0.001	<0.001
4	26.1^a^	31.1^b^	0.47	<0.001	0.01
6	25.8^a^	35.1^b^	1.51	0.01	0.01
8	27.2^a^	30.8^b^	0.75	0.01	0.80
12	30.3^a^	32.1^a^	0.89	0.15	0.76
24	44.1^a^	41.8^b^	0.63	0.03	0.20

Data are presented as LSMEANS ± SEM. PCF, pea coarse flour, obtained with a crushing roller with a 2.5 mm space; PFF, pea fine flour, obtained with hammer mill with a 2 mm screen; *F*, feed effect (coarse vs. fine). Mean values in the same row without a common superscript are significantly different at *P* < 0.05.

The concentration of ammonia was lower for the PCF medium than that for the PFF until 6 h and then was higher after 8 and 12 h of incubation. After 24 h, there was no difference between media.

The concentration in soluble carbohydrates was quite stable during the first 8 h of incubation for the PCF medium and increased afterward. It was quite high for the PFF medium after 1 h of incubation, decreased during the following 3 h, and finally increased after 12 h of incubation.

### In situ degradation

The mean dry matter and CP degradation values for the PCF sample were significantly lower than those of the other samples, except after 48 h of incubation for the dry matter (Table 6).

**Table 6:** In situ degradation kinetics of pea flours

							*P*-value				
							
Incubation time (h)	PCF		GPCF	PFF	GPFF	SEM	*F*	*G*	*F* × *G*	Animal	Run
Dry matter degradation (%)											
2	11.0^a^		68.7^b^	66.1^c^	69.3^b^	0.32	<0.001	<0.001	<0.001	0.20	0.12
4	13.4^a^		70.2^b^	68.3^c^	71.1^b^	0.47	<0.001	<0.001	<0.001	0.39	0.02
8	25.7^a^		76.4^b^	77.9^b^	77.6^b^	0.87	<0.001	<0.001	<0.001	<0.001	0.73
16	59.5^a^		85.4^b^	84.9^b^	86.2^b^	2.65	<0.001	<0.001	<0.001	<0.001	0.09
24	62.6^a^		90.5^b^	89.1^b^	93.6^b^	1.78	<0.001	<0.001	<0.001	<0.001	0.02
48	98.5		99.3	99.1	99.3	0.35	0.45	0.25	0.13	0.21	0.004
Model	1	2	1	1	1						
*a* (%)	0.5	11	64.5	62.1	64.4						
*b* (%)	121	89	39.1	39.6	38.0						
*c* (h^−1^)	0.034	0.054	0.0459	0.0529	0.0554						
*l* (h)		3.8									
RSD (%)	6.4	12.0	0.74	1.72	1.12						
ED (%)	44.3	44.5	81.4	80.7	82.6						
Crude protein degradation (%)											
2	5.5^a^		80.3^b^	75.1^c^	79.3^d^	0.26	<0.001	<0.001	<0.001	0.21	0.26
4	9.5^a^		80.9^b^	78.4^c^	81.0^b^	0.30	<0.001	<0.001	<0.001	0.68	0.30
8	21.5^a^		85.7^b^	86.2^b^	87.0^b^	0.63	<0.001	<0.001	<0.001	<0.001	0.63
16	59.5^a^		92.4^b^	90.7^b^	92.3^b^	1.88	<0.001	<0.001	<0.001	0.015	1.88
24	63.6^a^		95.7^b^	93.0^b^	97.4^b^	1.07	<0.001	<0.001	<0.001	<0.001	1.07
48	98.6		nd	nd	nd						
Model	1	2	1	1	1						
*a* (%)	−8.5	7.5	77.1	68.6	76.2						
*b* (%)	105.5	92.5	28.2	25.2	29.6						
*c* (h^−1^)	0.0525	0.062	0.046	0.1375	0.0519						
*l* (h)		5.1									
RSD (%)	6.61	11.8	0.783	0.744	0.707						
ED (%)	40.7	42.2	89.3	86.1	89.9						

For the fine flours, the degradation was described using only the Ørskov and McDonald model. For the coarse flours, degradation was described using both the Ørskov and McDonald and the McDonald models. Data are presented as LSMEANS ± SEM. PCF, pea coarse flour, obtained with a crushing roller with a 2.5 mm space; GPCF, PCF reground with a screen of 1 mm aperture; PFF, pea fine flour, obtained with hammer mill with a 2 mm screen; GPFF, PFF reground with a screen of 1 mm aperture; *F*, feed effect (coarse vs. fine); *G*, regrinding effect; *F* × *G*, interaction between feed and regrinding effects; ED, effective degradability; 1, model Ørskov and McDonald (1979); 2, model McDonald (1981) with constraint (*a* + *b*) ≤ 100. Mean values in the same row without a common superscript are significantly different at *P* < 0.05.

The *a* value for dry matter degradation was close to 0% for PCF versus 62% for PFF with the first model (Table 6) demonstrating a much higher degradation rate or short-term solubilization of PFF in comparison with PCF. As a consequence, the PCF exhibited a larger fraction *b* with a lower fractional degradation rate, and consequently a lower ED than PFF. The parameters for the ground samples (GPCF and GPFF) were similar to those of PFF. The ranking of the pea samples was the same for the ED of CP with a greater difference between PCF and the others. However, the high values of residual standard deviation or RSD (6.4 and 6.6) obtained for PCF reveal the difficulty in fitting curves that present a lag time at the start of degradation. Moreover, the *b* values obtained for PCF were greater than 100. This fact and the negative *a* value for CP degradation are due to the low degradation rates observed for this pea flour. As these values did not seem the most relevant, a second model was used with constraints on the *a* (≥0) and *b* (≤100) values for the dry matter and CP in situ degradations. It also included a lag time. The effective degradabilities were similar to those obtained with the first model. The in situ degradability was also calculated after 16 h 40 min duration. The values obtained were a little higher than for the dry matter ED: around 53% versus 44% for PCF, and between 85.3% and 87.3% versus 80.7% and 82.6% for the three other samples. The ranking of the samples was the same for the two methods.

### Comparison in vitro—in situ

**Figure 3:**
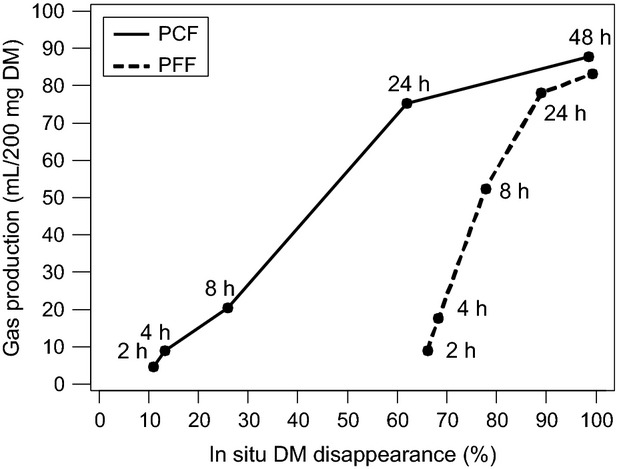
Relation between patterns of gas production and in situ dry matter degradation. PCF, pea coarse flour, obtained with a crushing roller with a 2.5 mm space; PFF, pea fine flour, obtained with hammer mill with a 2 mm screen.

**Figure 4:**
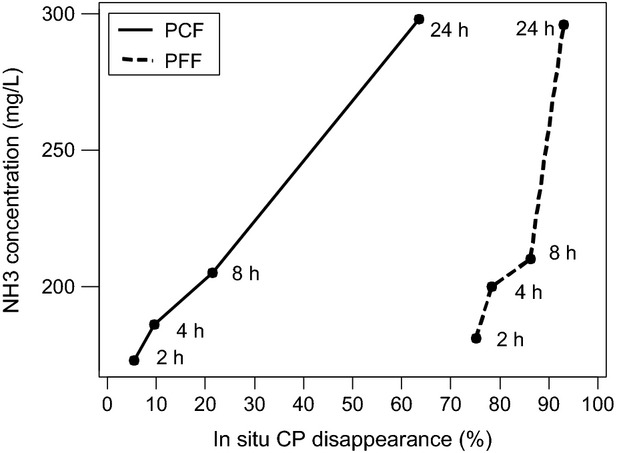
Ammonia production measured by in vitro gas method and in situ crude protein (CP) degradation. Values are plotted for matching timepoints. PCF, pea coarse flour, obtained with a crushing roller with a 2.5 mm space; PFF, pea fine flour, obtained with hammer mill with a 2 mm screen.

The in situ, DM, and CP effective degradabilities and the in vitro GP16 exhibited a quite similar ranking of the four grinding protocols. The degradation kinetics of pea flours obtained in vitro and in situ are shown in Figures 3, 4.

After 24 h of incubation, the gas production and the in vitro ammonia concentrations were similar for both pea flours, but not the in situ DM or CP disappearances. The amount of gas produced per gram DM degraded in situ was higher for PCF than that for PFF: 600 versus 438 mL gas g^−1^ DM disappeared. Similar differences were seen in NH_3_ in vitro concentration per mg of in situ CP disappearance PCF: 9.60 versus PFF: 6.71 g L^−1^ NH3 g^−1^ CP. However, looking at the disappearance between 2 and 24 h of fermentation, the ratio of gas production and in situ DM degradation was lower for PCF than for PFF (683 vs. 1504 mL gas g^−1^ DM disappeared). Similar results were obtained for the increase in NH_3_ in vitro concentration between 2 and 24 h of incubation per mg of in situ CP disappearance PCF: 22.7 versus PFF: 73.7 g L^−1^ NH3 g^−1^ CP.

### Nutritive value of the pea flours

#### Energy value

The organic matter digestibility (OMD) of the feeds can be predicted with an equation integrating gas production after 24 h of incubation (mL), CP, and ash contents according to Menke and Steingass (1988). The estimated OMD values were of 92.5% for PCF and 94.0% for PFF (SEM = 0.41). The difference between the flours, tested according to the Bonferroni test, was not statistically significant.

The gas production in the short and medium terms was significantly lower with the PCF flour compared to the PFF, in agreement with the in situ dry matter degradation differences. This means that the degree of acidogenicity of the PCF sample is lower than that of the PFF one.

#### Nitrogen value

The pea PDI values were estimated from the in situ measurements, according to Vérité et al. (1987). The PDIA (PDI brought by the feed) fraction that corresponds to the part of the feed that resisted ruminal degradation is estimated from the CP content and nitrogen ED. It differed a lot between feeds: 132 g kg^−1^ DM for PCF versus 29 g for PFF. Accordingly, the N source available for the microbial population was lower for PCF compared to PFF: the PDIMN (protein digestible in the small intestine supplied by microbial protein from rumen-degraded dietary protein) values were, respectively, 56 g kg^−1^ DM for PCF and 118 g for PFF. The PDIME (protein digestible in the small intestine supplied by microbial protein from rumen-fermentated organic matter) values corresponding to the energy source available for the rumen microbes were calculated from the estimated rumen fermentable organic matter (FOM), which were lower for PCF than those for PFF (respectively 735 and 856 g FOM kg^−1^ DM). The PDIME value of PCF was lower than that of PFF (68 vs. 79 g kg^−1^ DM). Thus, PFF degradation seemed potentially to bring more N than carbohydrates to ruminal microbes and in contrast, PCF degradation provides more carbohydrates than N. Finally, PCF values of PDI were higher than those of PFF ones, with respectively 188 and 147 g PDIN kg^−1^ DM and 200 and 108 g PDIE kg^−1^ DM. The limiting PDI was PDIN for PCF and PDIE for PFF.

## Discussion

The grinding processes induced a clear separation between the two pea flours from a granulometric point of view and allowed us to separate the effects due to the physical characteristics from the chemical ones. The CP contents of peas were quite similar, as was ash content. Nevertheless, the PFF had lower cell wall contents than the coarse one. Some particles might have been lost during the grinding process or losses of feed occurred during the analysis with the Van Soest method, which is a gravimetric one. The difference in buffering capacity between PCF and PFF can be partly explained by the difference in specific surface area. An increase in the surface area corresponded to an increase in the buffering capacity of feeds. This explanation was confirmed by the buffering capacity of the ground coarse pea flour (GPCF), which is similar to the PFF one.

The differences in the fermentation measurements observed with the PCF compared to the PFF were only due to the particle size as the GPCF did not differ from PFF. The PFF flour had a very high fermentation activity in the first hours of incubation. This could be easily explained by the high specific surface area of PFF, which corresponded to the surface per mass unit which would be available to microorganisms (Bjorndal et al. 1990; Richards et al. 1995). The easier access to the cellular constituents by the rumen microflora led to a faster acidification and a lower pH (Gerson et al. 1988). Notwithstanding the rate of acidification of the medium was faster for PFF than for PCF, the pH was always between 6.62 and 6.89, that is higher than 6.2 considered as a threshold below which Menke's buffer is exhausted and the production of gas is not linearly correlated with the formation of end-products (Tagliapietra et al. 2011).

The in vitro results of NH_3_ concentration confirmed that pea proteins are highly soluble (Michalet-Doreau 1992; Guedes and da Silva 1996), especially for the PFF flour, because the grinding process had increased the availability of soluble nutrients by breaking the cell wall structures (Lambert et al. 1998; Wadhwa et al. 1998). With PFF there was a high rate of NH_3_ production in the short term, followed by a transient decrease which could be explained by pea starch availability: pea starch consists of about 400 g kg^−1^ of amylose and of 600 g kg^−1^ of amylopectin, which are not degraded at the same rate (Ratnayake et al. 2002). Zhou et al. (2004) showed that starch exhibited a biphasic hydrolysis pattern with a relatively rapid rate initially, followed by a lower rate thereafter. During this phase of intense fermentation, NH_3_ might have been recaptured by microorganisms for their growth (Chen et al. 1987). With the same in vitro method, a transient decrease in ammonia nitrogen concentration was also observed by Guzzon et al. (1997) during the period of active fermentation of cellulose and maize starch. This role for ammonia of intermediate substrate between degradation and assimilation of nitrogen by microorganisms was also emphasized by Al-Rabbat et al. (1971) and Tamminga (1979). It could be also modeled, as shown by the mechanistic model proposed on pea seed fractions data by Serment and Sauvant (2010). The rate of NH_3_ production was more constant and lower for the PCF flour, which degraded more slowly.

The evolution in the concentration of soluble carbohydrates can be explained by the balance between degradation of carbohydrates and their utilization by microbes. With the PFF sample, soluble carbohydrates were released very rapidly but used more quickly (Hindle et al. 2005; Yang et al. 2005) even if a delay of 1.5 h before their utilization by microorganisms has been reported (Sauvant and Van Milgen 1995). This assumption is in agreement with the values observed with the PFF flour after 1 and 2 h of incubation. As in our trial, Cone et al. (1997) observed that in the incubation medium, as gas production stabilized, the energy store (glucose) and the microbial population (microbial nitrogen) decreased and the amount of NH_3_ progressively increased, likely acting in the long term as a marker of microbial lysis in syringe.

This study confirmed that a decrease in particle size induced an increase in the in situ degradation of pea. For CP, Michalet-Doreau and Cerneau (1991) indicated that the ED decreased from 94.7% to 82.4% as mean particle size increased from 186 to 1032 *μ*m. When various data in the literature about protein effective degradation of protein rich feed were pooled (Freer and Dove 1984; Michalet-Doreau and Cerneau 1991; Maaroufi et al. 2009), it appeared that an increase of 1000 *μ*m of mean particle size induced a mean decrease of 14.1 ± 1.00% of the protein ED, lower than the 18.3% found in this study.

The in situ results of the fine flours were very close to those of the pea seed in the INRA-AFZ feed table (Sauvant et al. 2004) for the *a* and *b* fractions and for the ED. This is because the in situ parameters given in the table were obtained on fine pea flours. The difference between the PCF flour and the fine flours is in agreement with the data of Bayourthe et al. (2000) who compared the effect on pea of several grindings, and in particular two samples, the mean diameter of which were 2025 nm and 267 nm, comparable to flours used in this study.

It must be kept in mind that there was a high loss of pea particles through the nylon bag as measured by Michalet-Doreau (1990) on dry matter (16.5% loss) or nitrogen (21.5%) or as disappearance after 2 h of incubation (71.9% for DM and 78.8% for N). As microbial digestion leads to a decrease in particle size, small particles may escape bags as time progresses (Ehle et al. 1982; Bowman and Firkins 1996) and thus the in situ method underestimates the proportion of protein that escapes ruminal degradation. The in situ method assumes that solubility or disappearance is equivalent to degradability.

However, NH_3_ patterns with the in vitro method showed that in the case of the pea, even if finer grinding led to an important in situ CP disappearance, the NH_3_ produced was better captured by the microbial population than was the case for coarser grinding (Maaroufi et al. 2009).

The comparison of the in situ and in vitro methods confirmed the overestimation of in situ degradation (Lopez et al. 1998). In fact, the DM and CP initially disappearing from the bags were at least 60% for the PFF, whereas the gas production was almost null with in vitro incubations. These effects were minimized with a coarser grinding.

The nutritive value of PCF differed from that of PFF. PCF degradabilities were lower than PFF ones. The estimation of the OMD by the in vitro method was overestimated for the PFF flour when compared with the values proposed in the 2004 INRA-AFZ tables (92%; Sauvant et al. 2004) but agreed with those for the PCF flour. It must be kept in mind that the feed sample has to be ground at a 1 mm screen in the in vitro method what is similar to the PFF flour. Besides, Abreu and Bruno-Soares (1998) underlined that in vitro gas production is not a good prediction criterion of in vivo OMD when dealing with legume grains.

Compared to PCF, the PFF flour presents a greater risk of inducing acidosis because a higher rate of degradation induces a greater pH decrease and animals spend less time chewing fine particles (Sauvant et al. 2006). Moreover, with the PFF medium, there was also a greater ammonia concentration, which means that nitrogen not used by microbes could be lost into urine. Thus, coarse particles could be of better nutritive value and safer than fine ones as shown by the results obtained with both methods.

## Conclusion

A coarse grinding of pea led to a lower ruminal degradation than a fine one. In this study, the only factor of variation was the grinding method of pea. The in situ method showed a high sensitivity to variations in granulometry that could lead to miscalculation of the protein value of pea by feed evaluations based only on in situ data. These in vitro and in situ results should be used in conjunction with in vivo performances using rations containing coarsely or finely ground pea flour to assess the practical influence of particle size on the nutritive value of pea protein for ruminants.
